# Post-pandemic insights on COVID-19 and premature ovarian insufficiency

**DOI:** 10.1515/biol-2022-1028

**Published:** 2025-01-28

**Authors:** Yaguang Han, Yang Dai, Kexin Wang, Xin Zhang, Zishen Shao, Xiaolin Zhu

**Affiliations:** Department of Medicine, First Affiliated Hospital of Heilongjiang University of Chinese Medicine, Harbin, 150040, China; Department of Medicine, Second Affiliated Hospital of Heilongjiang University of Chinese Medicine, No. 411 Guogeli Street, Nangang District, Harbin, Heilongjiang, 150006, P.R. China

**Keywords:** COVID-19, premature ovarian insufficiency, immune dysregulation, clinical prevention, public health

## Abstract

The COVID-19 pandemic has raised concerns regarding its potential impact on premature ovarian insufficiency (POI). This overview examines the possible interactions between COVID-19 and POI, while also suggesting preventive measures. The viral infection’s inflammatory response and immune dysregulation may adversely affect ovarian tissues, leading to inflammation and damage. Additionally, alterations in vascular function could impair ovarian blood flow and hormonal imbalances may disrupt normal ovarian function. Long-term health effects, such as “long COVID,” may exacerbate these issues through chronic inflammation and immune dysfunction. Public health measures, such as vaccination and home isolation, may indirectly protect ovarian health by reducing systemic inflammation. Vaccines could mitigate the severity of COVID-19’s impact on ovarian function, while isolation may reduce stress and inflammation. However, further research is needed to validate these mechanisms.

## 1 Introduction

The COVID-19 pandemic, caused by the SARS-CoV-2 virus, has had a significant impact on global health. Extensive research has been conducted to examine its effects on various organs. However, its potential impact on reproductive health, particularly ovarian function and premature ovarian insufficiency (POI), remains inadequately explored. POI, characterized by the cessation of normal ovarian activity before the age of 40, can lead to infertility, hormonal imbalances, and an increased risk of osteoporosis and cardiovascular disease [1].

COVID-19 triggers robust inflammatory and immune responses, which may directly and indirectly affect ovarian health [2]. Systemic inflammation associated with COVID-19 involves a massive release of pro-inflammatory cytokines, known as a cytokine storm [3]. This heightened inflammatory state can extend to various organs, including the ovaries, potentially leading to tissue damage [4]. Ovarian tissues are sensitive to inflammatory insults, which can disrupt the delicate processes of folliculogenesis – the development and maturation of ovarian follicles necessary for ovulation [5]. Prolonged inflammation could result in a reduction of the ovarian reserve, the number of viable eggs within the ovaries, ultimately impairing overall ovarian function [6]. The immune dysregulation caused by COVID-19 may further complicate ovarian health [7]. The virus can disrupt the balance of the immune system, occasionally triggering autoimmune reactions. Autoimmune oophoritis, where the immune system mistakenly attacks ovarian tissue, is a recognized cause of POI. In the context of COVID-19, the hyperactivity of the immune system may similarly target ovarian tissues, leading to inflammation, follicular destruction, and compromised ovarian function [7].

Additionally, vascular complications associated with COVID-19, such as endothelial dysfunction and thrombosis, could disrupt blood flow to the ovaries [8]. The endothelium, the inner lining of blood vessels, can be severely affected by the virus, leading to dysfunction and an increased risk of clot formation [9]. Adequate blood flow is crucial for delivering oxygen and nutrients to ovarian tissues [10]. Any impairment in vascular function could result in hypoxia and nutrient deprivation, further compromising ovarian health and function.

Endocrine disruptions linked to COVID-19 might also affect ovarian hormone regulation [11]. The hypothalamic–pituitary–ovarian (HPO) axis, which regulates reproductive hormones, can be influenced by systemic diseases like COVID-19 [12,13]. Disruptions in this axis may alter the secretion of hormones such as gonadotropins (LH and FSH), estradiol, and progesterone, all of which are essential for regular menstrual cycles and ovarian function. These hormonal imbalances can lead to menstrual irregularities, anovulation, and a reduced ovarian reserve, thereby affecting fertility [14].

The phenomenon of “long COVID,” characterized by persistent symptoms and chronic inflammation, poses additional risks to ovarian health [15]. Long-term inflammation and immune dysregulation can create an environment of continuous stress on ovarian tissues. Chronic exposure to inflammatory cytokines and immune cells can lead to sustained ovarian damage, fibrosis, and even premature ovarian failure [16]. Long COVID may also exacerbate endocrine dysfunctions, further disrupting the HPO axis and ovarian hormone regulation, potentially leading to lasting damage to ovarian function. Public health measures, including vaccination campaigns and social distancing policies, may indirectly influence ovarian health [17]. Vaccination can mitigate systemic inflammation, while social distancing can reduce stress-related impacts on reproductive health [18]. However, these interventions must be balanced to protect both overall and reproductive health effectively.

Despite these considerations, the relationship between COVID-19 and POI remains speculative due to the limited direct evidence available. Comprehensive research is needed to clarify the mechanisms by which COVID-19 may affect ovarian function and to develop strategies to mitigate these effects. This review seeks to synthesize current knowledge regarding the potential interactions between COVID-19 and ovarian function, highlighting the necessity for further investigation to guide clinical practices and safeguard women’s reproductive health during and after the pandemic.

## 2 Impact of COVID-19-induced inflammation on ovarian health and POI

Following COVID-19 infection, the body initiates a cascade of inflammatory and immune responses, including the release of cytokines, leading to what is referred to as a “cytokine storm.” This systemic inflammatory response may have both direct and indirect effects on ovarian health [19]. First, inflammatory mediators may indirectly affect ovarian tissue through the bloodstream, initiating inflammatory responses that result in subsequent damage to the ovarian tissue [20]. The ovaries, being metabolically active organs, are particularly sensitive to tissue inflammation, which can lead to abnormal follicle development and depletion, thereby affecting ovarian reserve and function. Second, COVID-19-induced immune dysregulation may exacerbate this process. The virus can disrupt immune system balance, potentially triggering autoimmune reactions [21]. Autoimmune ovarian inflammation is a recognized cause of POI, in which the immune system erroneously targets ovarian tissue as though it were a foreign invader [22]. In the context of COVID-19 infection, the overactivity of the immune system may similarly lead to inflammation and damage to ovarian tissue [23].

Interventions targeting these mechanisms can be implemented through various approaches. First, controlling and alleviating systemic inflammation is crucial. By reducing the severity of the cytokine storm, direct damage to ovarian tissue caused by inflammation can be minimized. Anti-inflammatory drugs may provide a potential solution, although their use during pregnancy necessitates careful consideration [24]. Regulating the activity of the immune system is also important [25]. Modulating the immune response following viral infection to prevent overactivation may help mitigate the development of autoimmune ovarian inflammation. Ensuring an adequate blood supply to ovarian tissue is also crucial. By preserving vascular function and preventing clot formation, sufficient delivery of oxygen and nutrients to the ovaries can be maintained, thereby minimizing tissue damage.

**Figure 1:**
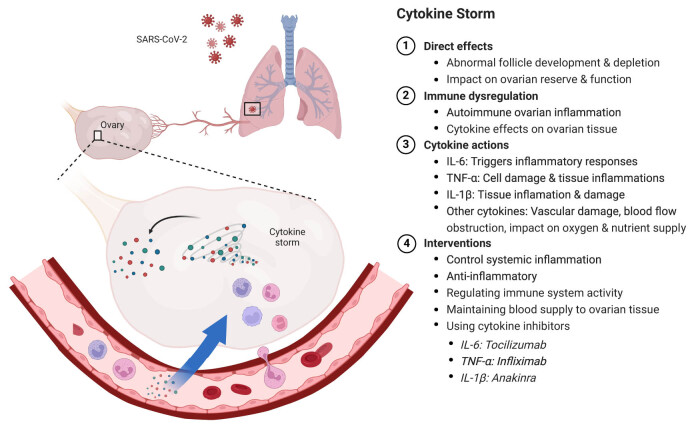
Effects of COVID-19 on the ovarian immune microenvironment. When COVID-19 infection occurs, the body triggers a cascade of inflammatory and immune responses, including a “cytokine storm,” which can directly and indirectly impact ovarian health. Inflammatory mediators may reach ovarian tissue through the bloodstream, causing damage due to inflammation. The highly metabolically active ovaries are sensitive to such inflammation, potentially leading to abnormal follicle development and decreased ovarian reserve. Additionally, COVID-19-induced immune dysregulation can exacerbate this process, potentially leading to autoimmune ovarian inflammation, a known cause of POI. To mitigate these effects, interventions may focus on controlling systemic inflammation, regulating immune activity, and maintaining good blood supply to ovarian tissue. Various cytokines, including IL-6, TNF-α, and IL-1β, among others, play roles in the inflammatory response and can affect ovarian health by causing tissue damage, apoptosis, oxidative stress, and vascular issues. Specific inhibitors or antagonists targeting these cytokines, such as IL-6 inhibitors (e.g., tocilizumab), TNF-α antagonists (e.g., infliximab), and IL-1β inhibitors (e.g., anakinra), may be utilized to reduce inflammation and protect ovarian tissue.

The inflammatory and immune responses triggered by COVID-19 involve various cytokines and immune cells, including but not limited to interleukin (IL)-6, tumor necrosis factor-alpha (TNF-α), IL-1β, IL-8, IL-10, IL-12, IL-17, IL-23, and interferon-gamma (IFN-γ) [26–29]. IL-6, as a key pro-inflammatory cytokine, may trigger inflammatory responses in ovarian tissue [30]. TNF-α’s excessive release may lead to cell damage and tissue inflammation, affecting ovarian health [31]. Aberrant expression of IL-1β may result in tissue inflammation and damage, further impacting ovarian tissue [32]. Additionally, other pro-inflammatory cytokines such as IL-8, IL-10, IL-12, IL-17, IL-23, and the anti-viral cytokine IFN-γ, may also influence ovarian health through different pathways [33]. The excessive release of these cytokines can lead to cell and tissue damage in the ovaries, potentially inducing apoptosis, oxidative stress, and inflammatory responses. Additionally, they may affect ovarian vascular permeability and stability, resulting in vascular damage, blood flow obstruction, and, consequently, impairing the supply of oxygen and nutrients to ovarian tissue. Controlling these cytokines may involve the use of specific inhibitors or antagonists targeting them. For instance, drugs such as IL-6 inhibitors (tocilizumab), TNF-α antagonists (infliximab), and IL-1β inhibitors (anakinra) may be used to alleviate inflammation and protect tissue health [34,35]. Figure 1 shows the impact of Covid-19 on the ovarian immune microenvironment.

## 3 Understanding the vascular impact of COVID-19 on ovarian health and POI

In addition to triggering inflammation and immune responses, COVID-19 infection may also lead to various vascular complications. The virus can induce thrombosis and vascular dysfunction, which may adversely affect ovarian health. A significant association exists between COVID-19 infection and thrombosis, with some patients developing abnormal blood clot formation, leading to thrombotic complications such as deep vein thrombosis (DVT) and pulmonary embolism (PE) [36,37]. Thrombus formation could lead to insufficient blood supply to the ovaries. As highly metabolically active organs, the ovaries require an adequate supply of oxygen and nutrients to maintain their function [38]. Insufficient vascular supply may damage ovarian tissue, impairing follicular development and growth, ultimately leading to ovarian dysfunction. Furthermore, COVID-19 may induce vascular dysfunction. Studies have demonstrated that COVID-19 infection can cause endothelial cell damage and vascular dysfunction. Endothelial cells, which are critical components of the vascular wall, play a vital role in maintaining vascular function and blood circulation [39]. Endothelial cell damage triggered by COVID-19 may lead to abnormalities in vascular tone regulation, increased vascular permeability, and consequently affect ovarian blood supply and nutrient delivery [40]. These vascular complications could directly affect ovarian health and function. Thrombus formation and vascular dysfunction may lead to ovarian tissue ischemia and hypoxia, disrupting cellular metabolism and function within the ovaries [41].

In addition to affecting ovarian blood supply, these vascular complications may exacerbate ovarian dysfunction by disrupting hormonal balance within the ovaries. Endothelial cells not only regulate vascular tone and permeability but also play a role in the synthesis and release of hormones. Therefore, COVID-19-induced vascular damage may impair hormonal balance in the ovaries, further compromising reproductive function.

**Figure 2:**
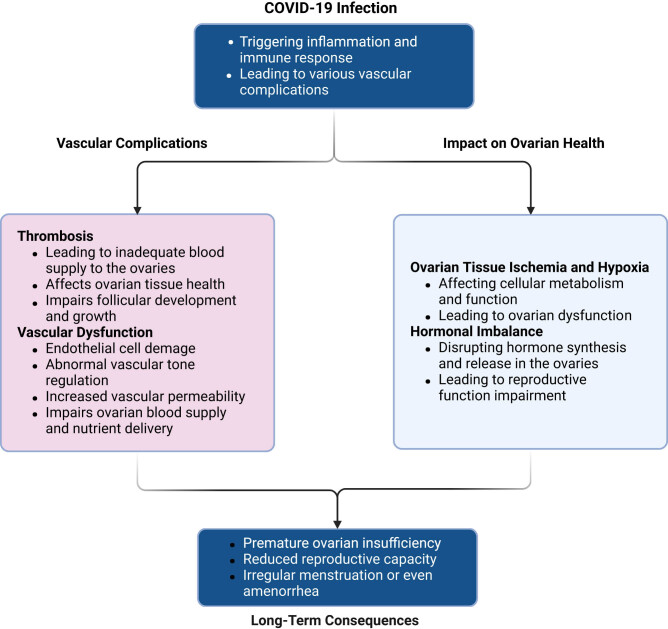
Impact of COVID-19-induced vascular complications on ovarian health. COVID-19 infection can lead to vascular complications, including thrombosis and endothelial dysfunction, which adversely affect ovarian health. The virus may induce abnormal blood clot formation, resulting in thrombotic events like DVT and PE, potentially leading to insufficient blood supply to the ovaries. As highly metabolically active organs, the ovaries require a robust blood flow for optimal function. Reduced vascular supply can damage ovarian tissue, impair follicular development, and cause ovarian dysfunction. Additionally, COVID-19 may cause endothelial cell damage, disrupting vascular tone regulation and increasing vascular permeability, thereby compromising ovarian blood supply and nutrient delivery. These vascular issues can result in ovarian tissue ischemia, hypoxia, and long-term consequences such as premature ovarian insufficiency (POI). Vascular complications may also affect hormonal balance within the ovaries, further impacting reproductive function. Preventive measures, including monitoring coagulation function, managing underlying conditions, and preventing thrombosis, are essential for protecting ovarian health.

Consequently, preventing and treating vascular complications is essential for protecting ovarian function. This includes regular monitoring of patients’ coagulation status, careful attention to vascular function, and the implementation of measures to prevent thrombosis. Moreover, actively managing underlying conditions in COVID-19 patients, such as hypertension and diabetes, may help reduce the incidence of vascular complications. The influence of COVID-19-induced ovarian vascular complications is depicted in Figure 2.

## 4 Interplay of COVID-19, endocrine dysregulation, and POI

COVID-19 is closely associated with endocrine dysregulation [42], potentially exerting adverse effects on ovarian function and consequently leading to POI. Endocrine dysregulation is a significant factor contributing to POI, potentially induced by the inflammatory and immune responses triggered by COVID-19, as well as its direct impact on the HPO axis [43].

COVID-19 infection may primarily trigger systemic inflammation and immune responses, which could directly impact the function of the hypothalamus and pituitary gland, thereby disrupting the normal regulation of the HPO axis [43]. The hypothalamus and pituitary gland act as central regulators of the endocrine system, and any dysfunction in these structures may result in irregular hormone levels, thereby impacting ovarian function [44]. This inflammation-induced endocrine dysregulation may intensify inflammatory responses in ovarian tissue, further compromising ovarian function. Moreover, the inflammatory and immune responses triggered by COVID-19 may directly damage ovarian tissue, impairing its sensitivity and responsiveness to hormones [45]. The ovaries, being highly metabolically active organs, are particularly sensitive to inflammation. Inflammatory states can interfere with follicular development and reduce ovarian reserve and function. These inflammatory mediators may directly impact ovarian tissue via the bloodstream, thereby exacerbating ovarian dysfunction [46].

The prolonged presence of COVID-19 further heightens the risk to ovarian health. Persistent inflammation and immune dysregulation may contribute to ongoing ovarian damage and premature ovarian failure. In this context, the ovaries may be subjected to prolonged inflammatory effects, leading to structural and functional impairment. Chronic immune dysregulation could exacerbate this process, increasing the ovaries’ vulnerability to damage and disrupting their normal physiological functions [47]. Such prolonged damage and dysfunction could have irreversible effects on reproductive capacity, potentially leading to reduced fertility or even complete infertility. Additionally, long-term COVID-19 may exacerbate endocrine imbalances, further compromising ovarian function. Instability within the endocrine system can cause abnormal fluctuations in hormone levels, disrupting normal ovarian processes such as follicular development and ovulation.

**Figure 3:**
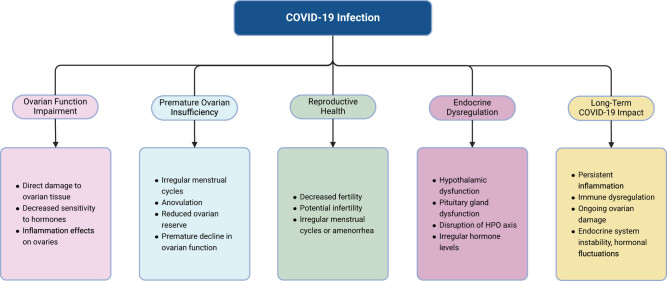
Impact of COVID-19-induced endocrine dysregulation on ovarian function. COVID-19 is associated with endocrine dysregulation, which can adversely affect ovarian function and potentially lead to premature ovarian insufficiency (POI). The infection may induce systemic inflammation and immune responses, disrupting the HPO axis and affecting the hypothalamus and pituitary gland, key regulators of the endocrine system. This disruption can lead to irregular hormone levels, further impacting ovarian function. Inflammatory responses triggered by COVID-19 can also directly damage ovarian tissue, affecting its sensitivity to hormones and impairing follicular development. Prolonged COVID-19 infection exacerbates these effects, potentially resulting in sustained ovarian damage, structural and functional impairment, and premature ovarian failure. Persistent immune dysregulation may increase susceptibility to ovarian damage, leading to decreased fertility, irregular menstrual cycles, and reduced ovarian reserve. The cumulative impact of these factors underscores the significant implications of endocrine dysregulation on women’s reproductive health.

Consequently, the endocrine dysregulation induced by COVID-19 may accelerate the onset of POI. This dysregulation may manifest as irregular menstrual cycles, anovulation, and reduced ovarian reserve, ultimately resulting in the premature decline of ovarian function. As ovarian function declines, fertility may diminish, menstrual cycles may become irregular or cease altogether, with significant implications for women’s reproductive health. Figure 3 illustrates the impact of COVID-19-related ovarian dysregulation.

## 5 Interplay between COVID-19 public health measures and ovarian health

Public health initiatives designed to curb the transmission of COVID-19, including extensive vaccination campaigns and social isolation policies, carry implications for ovarian health [11]. Vaccination against COVID-19 has emerged as a crucial strategy in combating the pandemic. Studies suggest that COVID-19 vaccines not only prevent severe illness and mortality but also alleviate the overall burden of the disease. By reducing the severity of COVID-19 infections, vaccines may indirectly contribute to the preservation of ovarian health [48]. COVID-19 is known to induce systemic inflammation, which can adversely affect ovarian function. Vaccination, by reducing the severity of the disease and mitigating inflammatory responses, may help protect ovarian function and reduce the risk of conditions such as POI. However, while vaccines may offer protective benefits, the potential negative effects on ovarian health warrant further investigation [49]. For instance, some individuals may experience transient side effects following vaccination, such as fever, fatigue, or muscle pain. While these side effects are typically mild and short-lived, their potential impact on ovarian function has not been extensively studied [50]. Additionally, there may be concerns about the long-term effects of COVID-19 vaccines on reproductive health, including ovarian function, fertility, and pregnancy outcomes [51].

Conversely, while long-term social isolation measures are essential for controlling viral transmission, they may have unintended consequences on reproductive health, including ovarian function. Prolonged periods of social isolation can lead to heightened psychological stress, disruptions in daily routines, altered sleep patterns, and changes in lifestyle factors such as diet and physical activity [52]. These stressors and lifestyle changes have the potential to disrupt hormonal balance and adversely affect ovarian function. Chronic stress can lead to dysregulation of the HPO axis, resulting in disturbances in menstrual cycles, ovulation, and overall ovarian function [13]. Additionally, changes in lifestyle habits during periods of isolation, such as increased sedentary behavior and poor dietary choices, may further exacerbate the risk of reproductive health issues.

**Figure 4:**
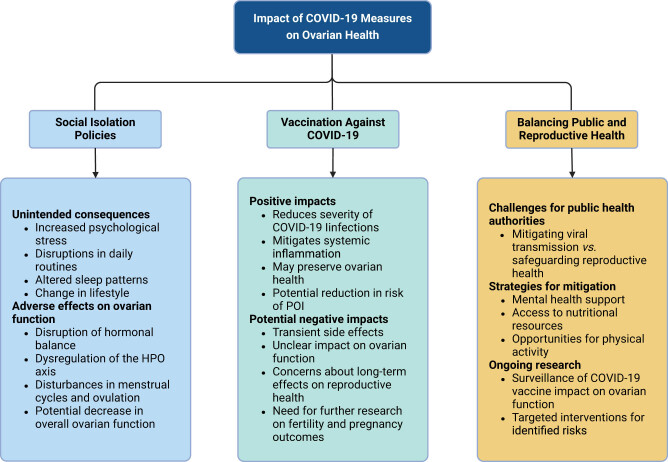
Impact of public health measures on ovarian health. Public health initiatives to control COVID-19, such as extensive vaccination campaigns and social isolation measures, have implications for ovarian health. Vaccination against COVID-19 plays a crucial role in reducing severe illness and disease burden. By minimizing the severity of infections and associated systemic inflammation, vaccines may indirectly protect ovarian function and reduce the risk of conditions like premature ovarian insufficiency (POI). However, potential short-term side effects of vaccination, such as fever and fatigue, and long-term impacts on reproductive health remain under investigation. Additionally, prolonged social isolation, while crucial for reducing viral transmission, can lead to increased psychological stress, lifestyle changes, and disruptions in hormonal balance, which may adversely affect ovarian function. Chronic stress and altered lifestyle habits during isolation can disrupt the HPO axis, impacting menstrual cycles and ovarian health. Balancing the need to control the virus with protecting reproductive health requires comprehensive strategies, including mental health support, nutritional resources, and physical activity. Ongoing research is essential to understand the full impact of these public health measures on ovarian health and to develop interventions to address any potential risks.

Striking a balance between mitigating viral transmission and safeguarding reproductive health presents a significant challenge for public health authorities. Strategies designed to promote overall health and well-being, including mental health support, access to nutritional resources, and opportunities for physical activity, are critical in alleviating the potential negative effects of social isolation on ovarian health. Moreover, continuous research and monitoring are essential to assess the impact of COVID-19 vaccines on ovarian function and to implement targeted interventions addressing any identified risks or concerns. Figure 4 illustrates the effects of COVID-19 public health measures on ovarian health.

## 6 Discussion

While elucidating the complex relationship between COVID-19 and ovarian health underscores the potential risks to women’s reproductive well-being, it is crucial to explore proactive measures to mitigate these effects. Gaining an understanding of the underlying mechanisms through which COVID-19 impacts ovarian function provides a foundation for developing targeted interventions aimed at preserving reproductive health amidst the challenges posed by the pandemic.

A key approach for intervention involves modulating the inflammatory response triggered by COVID-19 [53]. Given the pivotal role of systemic inflammation in causing ovarian damage, strategies aimed at attenuating the severity of the cytokine storm merit careful consideration. Although the use of anti-inflammatory agents during pregnancy requires thorough evaluation, they may offer potential in mitigating the detrimental effects of inflammation on ovarian tissue [54]. Furthermore, immune modulation offers a promising strategy for mitigating autoimmune reactions that could contribute to ovarian inflammation and dysfunction. When carefully managed under the supervision of healthcare professionals, immunosuppressive therapies may alleviate immune-mediated ovarian pathology.

Maintaining vascular integrity is another critical aspect of safeguarding ovarian health in the context of COVID-19-induced vascular complications. Diligent monitoring of coagulation parameters, along with close attention to vascular function, can help prevent thrombotic events that may compromise ovarian blood supply [55]. Furthermore, the proactive management of comorbidities that increase susceptibility to vascular dysfunction, such as hypertension and diabetes, is essential for reducing the incidence of vascular complications and mitigating their impact on ovarian function.

Addressing endocrine dysregulation induced by COVID-19 necessitates targeted interventions to restore hormonal balance within the ovaries. Strategies to address disruptions in the HPO axis may include pharmacological treatments specifically designed to correct hormonal imbalances [12]. Furthermore, lifestyle modifications that support hormonal balance – such as implementing stress management techniques, maintaining a balanced diet, and adhering to a regular exercise regimen – can serve as valuable complements to pharmacological treatments in the restoration of ovarian function.

Vaccination campaigns and social distancing policies must be carefully calibrated to achieve effective viral containment while safeguarding reproductive health. COVID-19 vaccination demonstrates potential in reducing systemic inflammation and lowering the risk of ovarian pathology. Nevertheless, ongoing surveillance is critical to detect and address any potential adverse effects of vaccination on ovarian health, necessitating close collaboration between public health authorities and reproductive health specialists.

Conversely, prolonged social isolation measures underscore the need for proactive strategies to mitigate their potential impact on ovarian health. Offering mental health support, access to nutritional resources, and opportunities for physical activity can help address the stressors associated with social isolation, thereby minimizing its detrimental effects on reproductive health [56]. Furthermore, implementing targeted interventions aimed at restoring hormonal balance and addressing lifestyle factors disrupted by social isolation may play a critical role in supporting ovarian health during the challenges posed by pandemic containment measures.

In conclusion, addressing the multifaceted relationship between COVID-19 and ovarian health necessitates a comprehensive approach. This includes the development of targeted strategies to mitigate the underlying mechanisms by which COVID-19 impacts ovarian function. Leveraging insights into the interplay between COVID-19 and ovarian health enables healthcare professionals to design proactive measures to safeguard reproductive health amidst the unprecedented challenges of the pandemic.
